# Supplementary material for: Evidence for divergent cortical organisation in Parkinson’s disease and Lewy Body Dementia
Source: Nat Commun. 2025 Nov 25;16:11623. doi: 10.1038/s41467-025-66783-9 (PMC12749319; doi:10.1038/s41467-025-66783-9)
Supplement: Supplementary file 1 — Supplementary Information [file 41467_2025_66783_MOESM1_ESM.pdf]

Divergent cortical reorganisation in Parkinson's and Lewy Body Dementias

Dr Angeliki Zarkali, Dr George Thomas, Ms Naomi Hannaway, Ms Ivelina Dobreva, Dr Melissa Grant Peters, Prof Mina Ryten, Prof Rimona S Weil PhD

Supplementary Information

Contents

Divergent cortical reorganisation in Parkinson's and Lewy Body Dementias 1

Supplementary Information 1

Supplementary Methods 3

MRI quality control 3

3T MRI processing 3

Disease Specific Gene Lists 4

URLs 5

Supplementary Results 6

Supplementary Table 1. Detailed results of cognitive assessments in the 3T cohort 6

Supplementary Table 2. Subgroups of Lewy Body Dementia (LBD) patients and their clinical characteristics (3T cohort) 7

*Supplementary Table 3. Demographics and clinical characteristics of replication 7T cohort*8

Supplementary Table 4. Percentage variance explained by partial least square (PLS) regression components 9

Supplementary Figure 1. Structural Connectivity Gradient 2 (SC-G2) alterations in Lewy Body Diseases 10

Supplementary Figure 2. Differences in gradient scores between different LBD subgroups and controls (HC). 11

Supplementary Figure 3. Differences in gradient scores between LBD and PD-NC participants. 12

Supplementary Figure 4. Differences in gradient scores between different LBD subgroups and Parkinson's patients with normal cognition (PD-NC). 13

Supplementary Figure 5. Differences in functional gradient scores between LBD, PD-NC and control (HC) participants 14

Supplementary Figure 6. Robustness of structural gradient alterations to different sparsity thresholds 15

Supplementary Figure 7. Differences in inter-regional differentiation between patients with Lewy Body Dementias (LDB), patients with Parkinson's and normal cognition (PD-NC) and controls (HC) – Replication using 7T Quantitative MRI 17

Supplementary Figure 8. Differences in composite gradient difference score between LBD subgroups. 20
Supplementary Figure 9. Structural gradient alterations in Lewy body disease are associated with regional gene expression patterns 21
References.. 23

Supplementary Methods

MRI quality control

All raw data were visually inspected, blinded to clinical data, to ensure appropriate brain coverage and to identify artefacts, e.g. motion. Further visual inspection was performed throughout the processing, including the final images produced. Only scans passing quality control for each modality were included.

rsfMRI

In addition to visual inspection, we adopted strict motion-control criteria for 3T rsfMRI, given susceptibility to motion artefact[1]. Specifically, participants were excluded if any of the following was met: 1) mean frame-wise displacement (FD) >0.3mm, 2) any FD >5mm, or 3) outliers >30% of the whole sample. FD was calculated using the MRI Quality Control tool (MRIQC)[2]. This led to 23 participants being excluded from functional connectivity analyses (11 PD-NC and 12 LBD), resulting to a total of 111 participants included in functional connectivity analyses (n=23 controls, n=35 PD-NC, and n=50 LBD).

7T cohort

Of 65 participants (n=24 controls, n=23 PD and n=20 LBD) with acquired MPM and MP2RAGE data, 8 participants (n=4 PD and n=4 LBD) were excluded due to significant artefact on the acquired multiparametric maps, and 1 LBD participant was excluded due to artefact on MP2RAGE. This resulted in a total of 58 participants (n=24 controls, n=19 PD-NC and 15 LBD) included. Additionally, of the included participants at 7T, 3 (n=1 control, n=2 LBD) did not have MTsat maps acquired, leaving n=23 controls, n=19 PD-NC and 13 LBD participants in MTsat analyses.

3T MRI processing

The Schaefer parcellation[3] was used to generate 200 cortical regions of interest (ROIs) by segmenting each participant's T1-weighted image.

Structural connectome construction

DWI weighted images were preprocessed using the standard pipeline as implemented in MRtrix3.0[4]. This involves denoising[5], removal of Gibbs artefacts[6], eddy-current and motion artefact correction[7] and bias field correction[8]. The raw T1-weighted images were then registered to the diffusion-weighted image (rigid, affine transformation) using Niftyreg[9]. Five tissue anatomical segmentation was performed in native DWI-image space using the 5ttgen script in MRtrix. Anatomically-constrained tractography was then performed with 10 million streamlines[10], using the iFOD2 algorithm[11] and dynamic seeding with streamlines truncated at the interface of grey-white matter. Spherical deconvolution informed filtering of tractograms (SIFT2)[12] algorithm was then applied to reduce biases. Resulting streamlines were used to construct the structural connectome, weighted by streamline count and a cross-sectional area multiplier[12].

Functional connectome construction

Each participant's rsfMRI data underwent standard pre-processing using fMRIPrep 23.2.0[13] which is based on Nipype 1.8.6. The first 4 volumes were discarded to allow for steady state equilibrium. For each participant, a reference volume was generated, using a custom methodology of fMRIPrep, for use in head motion correction. Head-motion parameters with respect to the BOLD reference (transformation matrices, and six corresponding rotation and translation parameters) are estimated before any spatiotemporal filtering using FSL mcflirt[14]. Distortion correction was performed using a TOPUP implementation[15]. The BOLD reference was then co-registered to the T1w reference using FreeSurfer mri_coreg followed by FSL flirt with the boundary-based registration cost-function. Co-registration was configured with six degrees of freedom[16].

Sources of spurious variance were removed through linear regression including the Friston-24 head-motion parameters (trans/rot x,y,z; first derivatives; and their squared terms) and the mean CSF, mean white-matter, and global signals, each with first derivative and squared terms (total = 36 regressors). This was followed by calculation of bivariate correlations and application of Fisher transform.

Functional connectivity between ROIs was quantified as the Pearson correlation coefficient between mean regional BOLD time series resulting to a 200x200 undirected weighted connectivity matrix.

Disease Specific Gene Lists

The following gene lists for Mendelian genes associated with Parkinson's and Alzheimer's disease were used. For Parkinson's disease, genes were derived from Blauwendraat et al[17] Table 1, retaining only genes with high or very high confidence for Parkinson's causation. For Alzheimer's disease, we filtered for genes associated with Alzheimer's disease and genetic association score of >0.6 in OpenTargets. For both lists, we excluded any genes for which gene expression data was not available in the Allen atlas[18].

**Parkinson's disease**	**Alzheimer's disease**
SNCA	PSEN1
PRKN	APP
PARK7	PSEN2
LRRK2	APOE
PINK1	ABCA7
POLG	SORL1
ATP13A2	GRIN1
FBXO7	CDK5
GBA	ADAM10
PLA2G6	ACE
VPS35	ACHE
DNAJC6	APH1B
SYNJ1	BCHE
VPS13C	GRIN2B

URLs

Abagen: https://zenodo.org/record/3726257#.XqmYWaj0lPY
BigBrainWarp: http://github.com/caseypaquola/BigBrainWarp.
Brain Space: https://github.com/MICA-MNI/BrainSpace
Brain Stat: https://brainstat.readthedocs.io/en/master/
fMRIprep: https://fmriprep.org/en/stable/
gProfiler: https://biit.cs.ut.ee/gprofiler/gost
hMRI toolbox: https://github.com/hMRI-group/hMRI-toolbox
MRtrix: https://www.mrtrix.org/
Niftyreg: https://github.com/KCL-BMEIS/niftyreg
Neuromaps: https://netneurolab.github.io/neuromaps/
Open Targets: https://platform.opentargets.org/
Revigo: http://revigo.irb.hr/
SPM12: www.fil.ion.ucl.ac.uk/spm

Supplementary Results

Supplementary Table 1. Detailed results of cognitive assessments in the 3T cohort

Cognitive task	HC<br>n=23	PD-NC<br>n=46	LBD<br>n=62	p-value
Attention				
Half Stroop naming				
Digit Span Backward	9.2 (2.1)	9.4 (1.8)	8.5 (2.6)	p<0.001[c]
Executive function				
Category fluency	19.6 (5.7)	21.0 (5.7)	12.8 (5.1)	p<0.001[a,c]
Half Stroop interference	35.3 (37.6)	26.8 (8.0)	53.6 (32.6)	p<0.001[a,c]
Language				
Letter fluency	18.3 (5.7)	17.3 (4.5)	12.9 (6.1)	p<0.001[a,c]
Graded naming task	25.2 (3.5)	25.8 (2.4)	21.3 (5.3)	p<0.001[a,c]
Memory				
Word recognition	24.4 (0.9)	24.7 (0.7)	22.7 (2.7)	p<0.001[a,c]
Logical Memory Delayed	15.7 (4.5)	15.8 (2.8)	9.1 (5.1)	p<0.001[a,c]
Visuospatial function				
Benton's Judgement of line orientation	26.6 (3.4)	25.6 (3.8)	22.6 (4.7)	p=0.006[a,c]
Hooper	24.5 (3.0)	25.6 (3.5)	19.7 (5.4)	p<0.001[a,c]

[a]: difference between LBD-HC, [b]: difference between PD-HC, [c]: difference between LBD-PD
HC: Healthy controls, PD-NC: Parkinson's disease with normal cognition, LBD: Lewy body dementia
Kruskal-Wallis (post hoc Dunn) test was used for between-group comparisons.

Supplementary Table 2. Subgroups of Lewy Body Dementia (LBD) patients and their clinical characteristics (3T cohort)

Characteristic	Parkinson's with mild cognitive impairment (PD-MCI) n=4	Parkinson's dementia (PDD) n=22	Dementia with Lewy Bodies (DLB) n=36	p-value
**Age**	**66.8 (8.3)**	**67.7 (6.8)**	**71.5 (6.3)**	**0.017**
**Male (%)**	**3 (75.0)**	**12 (63.2)**	**33 (91.7)**	**0.172**
**Right-handed (%)**	3 (75.0)	16 (84.2)	33 (91.7)	0.568
**Years of education**	16.8 (3.6)	17.3 (3.7)	16.8 (3.5)	0.289
**MOCA**	26.3 (5.3)	24.0 (6.0)	22.8 (5.3)	0.264
**MMSE**	**27.3 (1.5)**	**27.5 (3.5)**	**25.9 (3.6)**	**0.038**
**HADS anxiety**	7.3 (3.2)	8.1 (5.3)	5.8 (4.6)	0.086
**HADS depression**	6.8 (3.0)	6.3 (3.9)	5.9 (6.8)	0.658
**Composite cognitive score**	**-0.6 (0.8)**	**-1.2 (1.4)**	**-2.6 (1.9)**	**0.010**
**Years of diagnosis**	**8.8 (3.1)**	**6.2 (4.9)**	**2.09 (1.8)**	**<0.001**
**UPDRS total**	71.8 (14.0)	70.3 (30.0)	63.8 (29.7)	0.639
**UPDRS part 3**	38.5 (7.3)	33.7 (12.8)	31.8 (17.8)	0.633
**Timed Up and GO**	13.6 (6.7)	10.4 (3.8)	9.5 (4.3)	0.189
**Functional Assessments Questionnaire**	7.0 (7.3)	6.7 (5.3)	9.1 (6.7)	0.199
**UMPDHQ**	2..0 (4.0)	2.4 (3.4)	3.3 (3.0)	0.271
**RBDSQ**	5.5 (3.3)	6.4 (3.6)	7.6 (4.3)	0.228

All results shown as mean (standard deviation) except otherwise indicated.
Characteristics were compared used ANOVA (post hoc Tukey) for continuous normally-distributed variables and Kruskal Wallis (post hoc Dunn) for non-normally distributed variables.
HADS: Hospital Anxiety and Depression Scale, MMSE: Mini-Mental State Examination; MOCA: Montreal Cognitive assessment; RBDSQ: REM Sleep Behaviour Disorder Sleep Questionnaire; UMPDHQ: University of Miami PD Hallucinations Questionnaire; UPDRS: Movement Disorders Society Unified Parkinson's Disease Rating Scale.

Supplementary Table 3. Demographics and clinical characteristics of replication 7T cohort

Characteristic	Controls (HC) n=23	Parkinson's with normal cognition (PD-NC) n=13	Lewy Body Dementias (LBD) n=13	p-value
**Age**	67.7 (8.0)	64.5 (5.1)	72.1 (3.4)	**p=0.020** [c]
**Male (%)**	11 (47.8)	9 (47.4)	9 (69.2)	p=0.233
**Right-handed (%)**	20 (86.9)	13 (68.4)	13 (100)	p=0.557
**Years of education**	17.9 (2.2)	18.0 (2.6)	17.9 (3.9)	p=0.995
**MOCA**	28.5 (1.5)	28.8 (1.1)	22.5 (4.5)	**p<0.001**[a,c]
**MMSE**	29.2 (0.9)	29.1 (1.0)	25.5 (4.8)	**p=0.028**[a,c]
**HADS anxiety**	3.8 (3.2)	4.9 (3.4)	4.0 (3.8)	p=0.523
**HADS depression**	1.7 (2.2)	4.5 (2.9)	4.5 (4.0)	p=0.003[a,b]
**Years of diagnosis**	-	7.8	5.5	p=0.240
**UPDRS total**	-	52.9 (15.8)	54.5 (37.2)	p=1.000
**UPDRS part 3**	-	32.6 (19.8)	26.2 (20.0)	p=1.000
**Timed Up and GO**	-	9.2 (5.3)	9.3 (5.1)	p=0.292
**Functional Assessments Questionnaire**	-	1.6 (2.6)	5.5 (8.4)	**p<0.001**
**CAF**	-	1.0 (2.1)	1.5 (3.2)	p=0.230
**One Day Fluctuations Questionnaire**	-	0.7 (1.0)	1.4 (2.7)	p=1.000
**LEDD**	-	665.8 (278.4)	531.4 (368.9)	p=0.320
**UMPDHQ**	-	1.8 (2.9)	1.7 (2.6)	p=1.000

[a]: difference between LBD-HC, [b]: difference between PD-HC, [c]: difference between LBD-PD. In bold significant differences between LBD and PD. All results shown as mean (standard deviation) except otherwise indicated. Characteristics were compared used ANOVA (post hoc Tukey) for continuous normally-distributed variables and Kruskal Wallis (post hoc Dunn) for non-normally distributed variables.

CAF: Clinician Assessment of Fluctuations; HADS: Hospital Anxiety and Depression Scale, LEED: Levodopa Equivalent Dose; MMSE: Mini-Mental State Examination; MOCA: Montreal Cognitive assessment; UMPDHQ: University of Miami PD Hallucinations Questionnaire; UPDRS: Movement Disorders Society Unified Parkinson's Disease Rating Scale.

Supplementary Table 4. Percentage variance explained by partial least square (PLS) regression components

Component	Variance explained in LBD vs HC SC-G1 changes	Variance explained in gene expression
1	14.0	44.8
2	3.2	5.3
3	0.7	0.6
4	1.0	1.6
5	0.6	0.7
6	0.5	0.8
7	0.4	1.0
8	0.8	1.3
9	0.5	1.0
10	0.6	1.4

LBD: Lewy bodies dementia, HC: Healthy Controls, SC-G1: Structural connectivity gradient 1

Supplementary Figure 1. Structural Connectivity Gradient 2 (SC-G2) alterations in Lewy Body Diseases

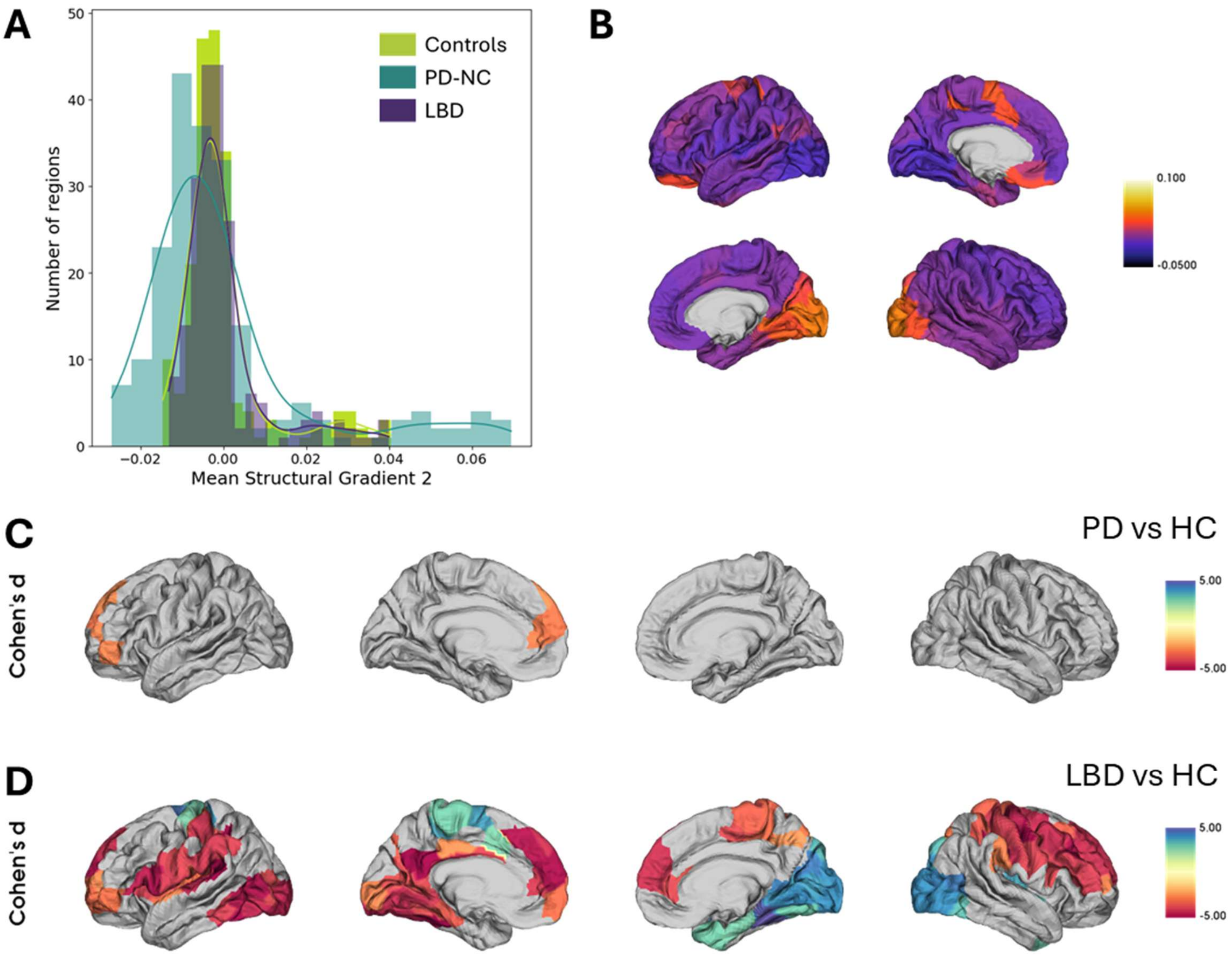

A. Overall gradient distribution in SC-G2 in controls (HC), Parkinson's patients with normal cognition (PD-NC) and patients with Lewy Body Dementias (LBD).

There was significantly different distribution amongst the three groups (Kruskal Wallis *H=17.00, p<0.001)* with expansion of gradient scores in PD-NC reflecting increased inter-regional differentiation in structural connectivity.

B. SC-G2 Gradient scores in healthy controls (n=23).

C. Differences in gradient scores between PD-NC and HC.

Surface-based linear models controlling for age and sex revealed significant differences in PD-NC vs HC with reductions in SC-G2 scores in left prefrontal regions temporopolar and dorsolateral prefrontal cortices. Only statistically significant clusters after multiple

comparisons correction (p_{FWE} <0.05) are shown. Colour scale depicts effect size per vertex (blue colours increases in gradient scores; red colours reductions in gradient scores).

D. Differences in gradient scores between LBD and HC.

LBD showed widespread changes in SC-G2 scores with both increases and reductions in scores reflecting widespread loss of cortical organisation. Only statistically significant clusters after multiple comparisons correction (p_{FWE} <0.05) are shown. Colour scale depicts effect size per vertex (blue colours increases in gradient scores; red colours reductions in gradient scores).

Supplementary Figure 2. Differences in gradient scores between different LBD subgroups and controls (HC).

Regional differences in Structural gradient 1 (SC-G1) scores between different subgroups and healthy controls (HC). The following subgroups were assessed separately:

- LBD: full subgroup of Lewy Body Dementia, as included in main results (n=62)
- DLB: patients with Dementia with Lewy bodies only (n=36)
- PDD: patients with Parkinson's dementia only (n=22),

Only statistically significant clusters after multiple comparisons correction (p_{FWE} <0.05) are shown. Colour scale depicts effect size per vertex (blue: decreases in scores; red: increases).

Overall SC-G1 distribution differed between LBD subgroups (Kruskal Wallis W=6.49, p=0.011) but this was driven by the PD-MCI group only (n=4) showing different distribution to DLB (p<0.001) with no other significant between sub-group differences.

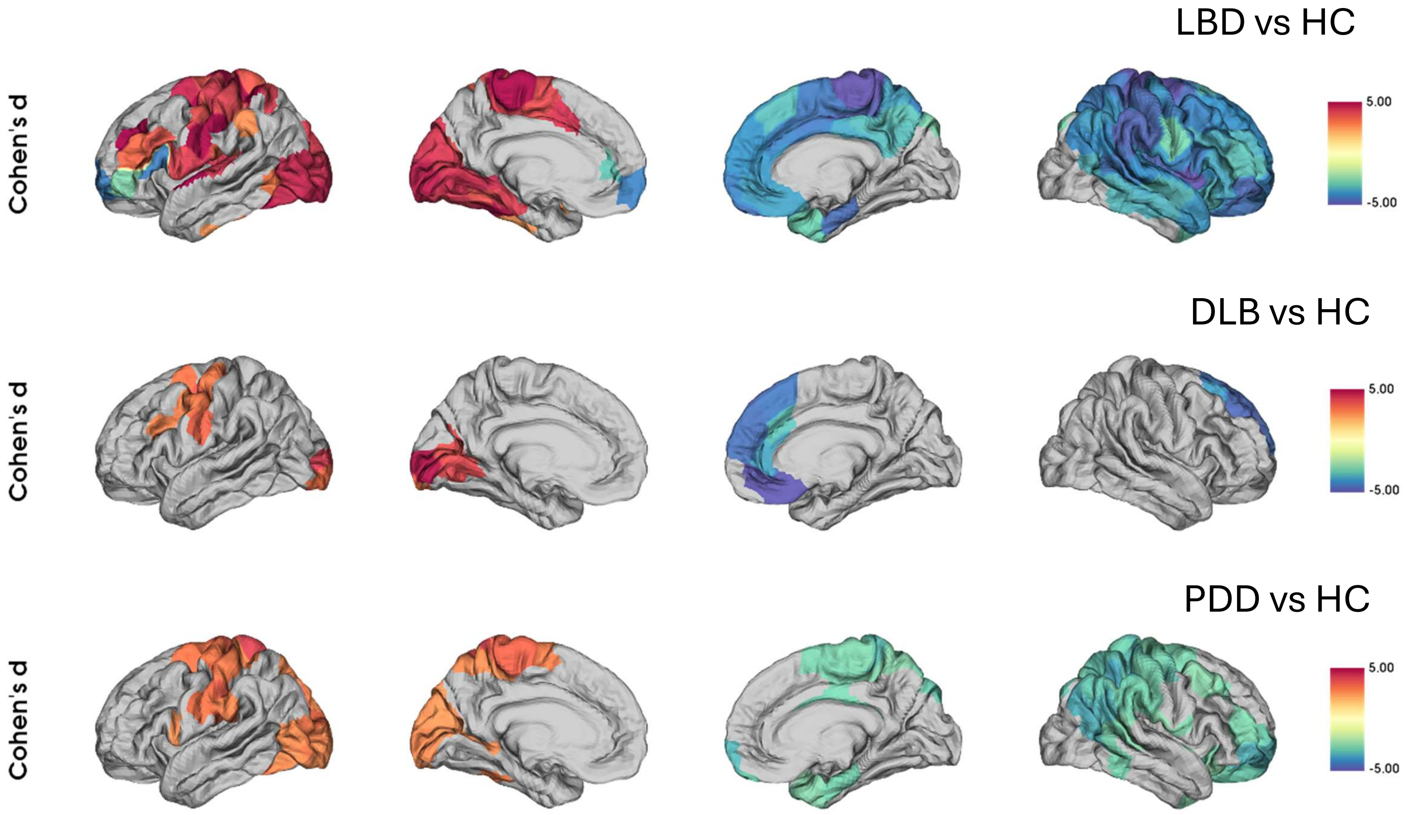

Supplementary Figure 3. Differences in gradient scores between LBD and PD-NC participants.

Surface-based linear models controlling for age and sex, using family-wise error correction (FWE) for multiple comparisons and cluster threshold 0.01 assessed differences between LBD (n=62) and PD-NC (n=46) participants. No significant differences were identified. Unthresholded, cluster-wise t-values are presented here for the two primary gradients. Colour scale depicts effect size per vertex (blue colours reductions in gradient scores; red colours increases in gradient scores).

Structural Connectivity Gradient 1 (SC-G1)

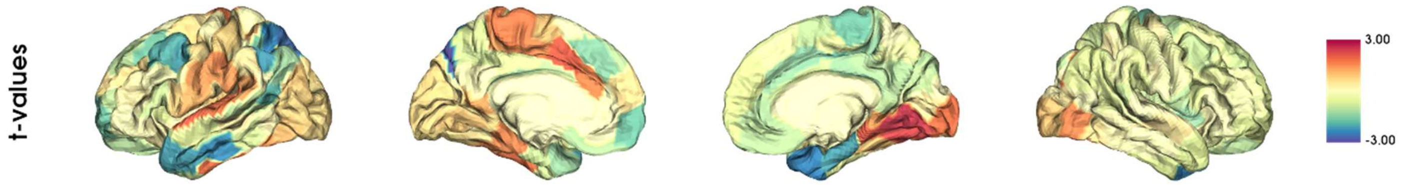

Structural Connectivity Gradient 2 (SC-G2)

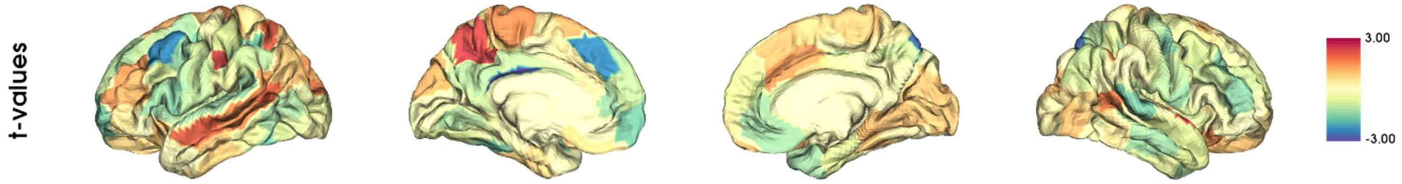

Supplementary Figure 4. Differences in gradient scores between different LBD subgroups and Parkinson's patients with normal cognition (PD-NC).

Surface-based linear models controlling for age and sex, using family-wise error correction (FWE) for multiple comparisons and cluster threshold 0.01 assessed differences in Structural gradient 1 (SC-G1) scores between different subgroups and PD-NC. The following subgroups were assessed separately:

- LBD: full subgroup of Lewy Body Dementia, as included in main results (n=62)
- DLB: patients with Dementia with Lewy bodies only (n=36)
- PDD: patients with Parkinson's dementia only (n=22),

There were no differences between LBD and HC (uncorrected results presented in Supplementary Figure 3), nor between PDD and PD-NC. There were significant differences between DLB and PD-NC. Importantly these were in the opposite direction than the differences seen between LBD and HC, PD-NC and HC, or DLB and controls; this suggests that this difference between DLB and PD-NC participants was not driving the results seen in our main analyses.

Colour scale depicts effect size per vertex (blue colours reductions in gradient scores; red colours increases in gradient scores)

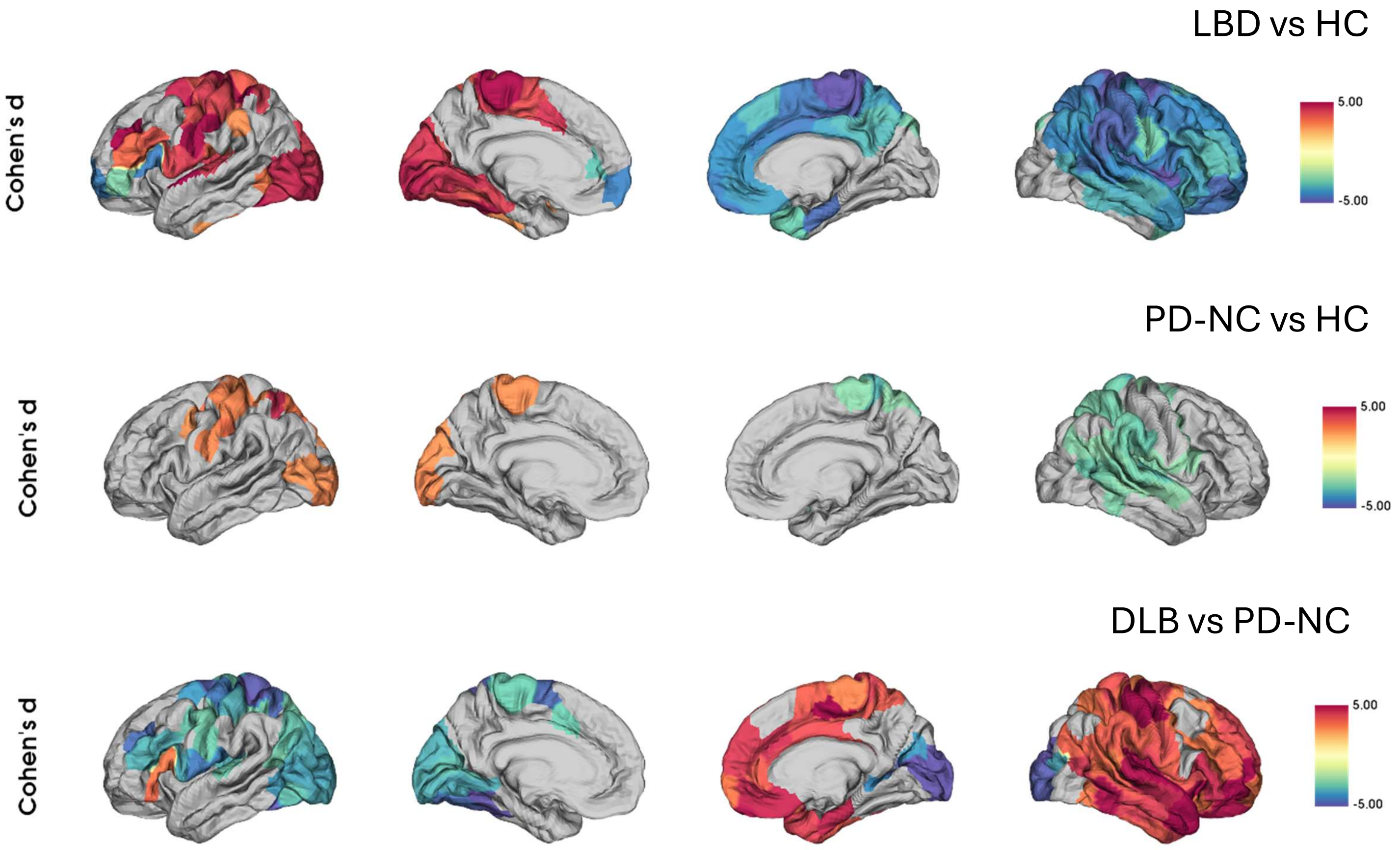

Supplementary Figure 5. Differences in functional gradient scores between LBD, PD-NC and control (HC) participants

In contrast to the pronounced differences in structural connectivity gradients shown in our cohort, functional connectivity gradients did not differ between any groups: Functional gradient 1 (Kruskal Wallis H = 0.018, p=0.991) and Functional gradient 2 (H=0.015, p=0.992). Additionally, there were no statistically significant differences at the vertex level between any two groups for either functional gradient. Surface-based linear models controlling for age and sex, using family-wise error correction (FWE) for multiple comparisons and cluster threshold 0.01 assessed differences between LBD (n=62) and PD-NC (n=46) participants.

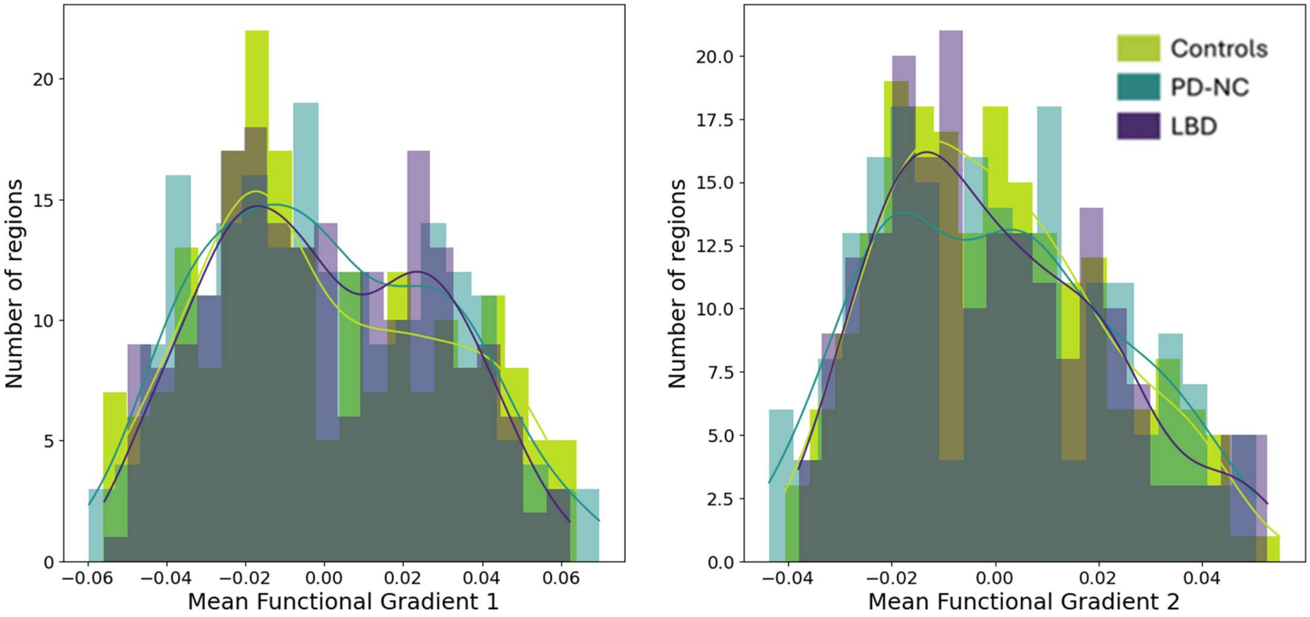

Supplementary Figure 6. Robustness of structural gradient alterations to different sparsity thresholds

We generated structural and functional gradients with different sparsity thresholds (0.8 and 0.5) to replicate our results.

A. The overall gradient distribution statistically differed between LBD, PD-NC and HC for both structural gradients 1 (SC-G1) and 2 (SC-G2) for both tested sparsity levels. The pattern of changes seen was also similar with gradient expansion in PD-NC (increased inter-regional differentiation) and apparent normalisation in LBD compared to HC.

B. Similar spatial distribution of regional changes in gradients between LBD vs HC and PD-NC vs HC for sparsity 0.8. **C.** Similar spatial distribution of regional changes in gradients between LBD vs HC and PD-NC vs HC for sparsity 0.5.

Surface-based mixed linear model was used with age and sex as covariates, FWE-correction for multiple comparison q<0.05, cluster threshold 0.01. Cohen's d for statistical significant vertices is presented (q<0.05) with red colours regions that have a lower gradient value in patients than controls and blue colours regions that have a higher gradient value

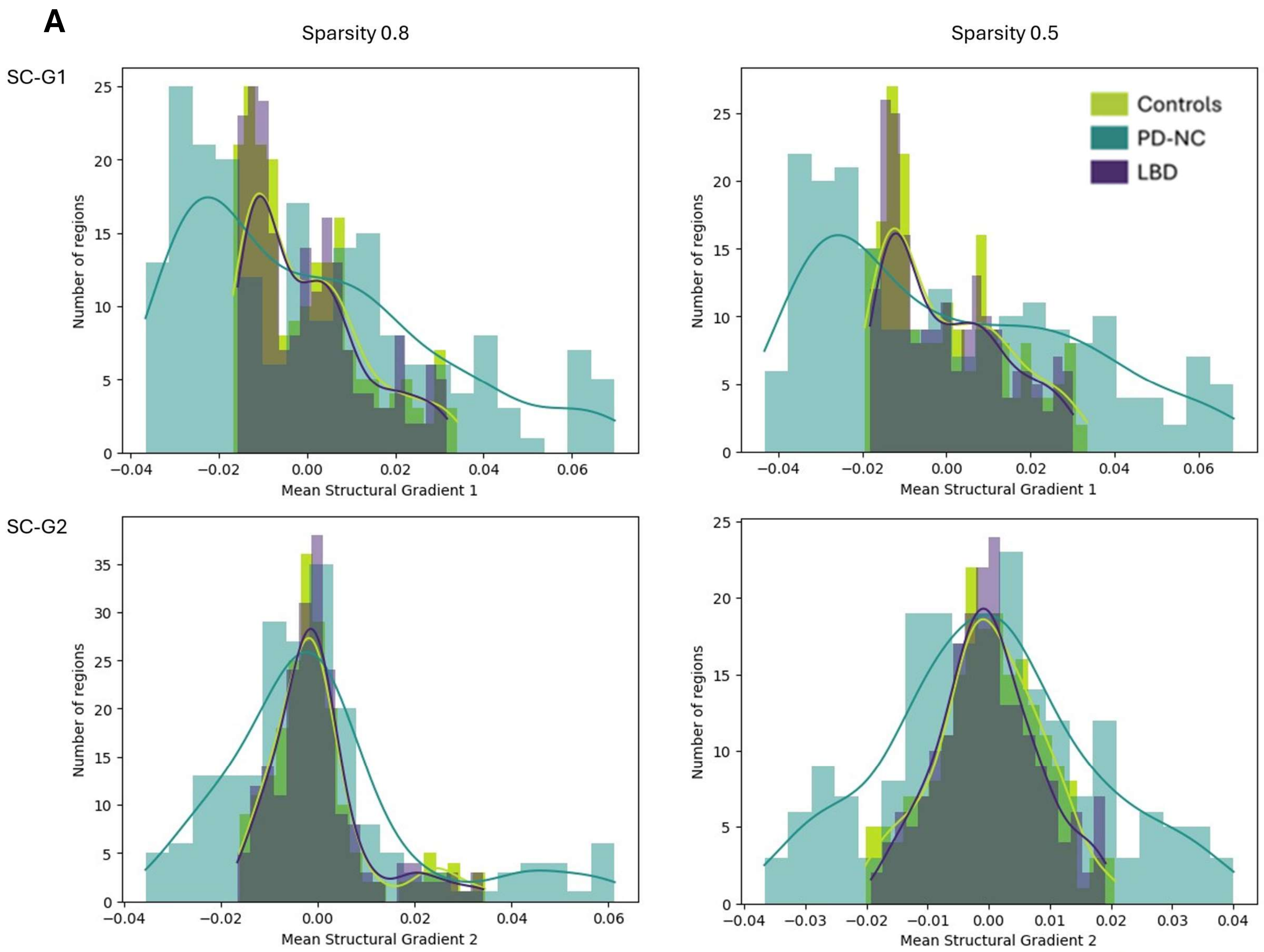

B

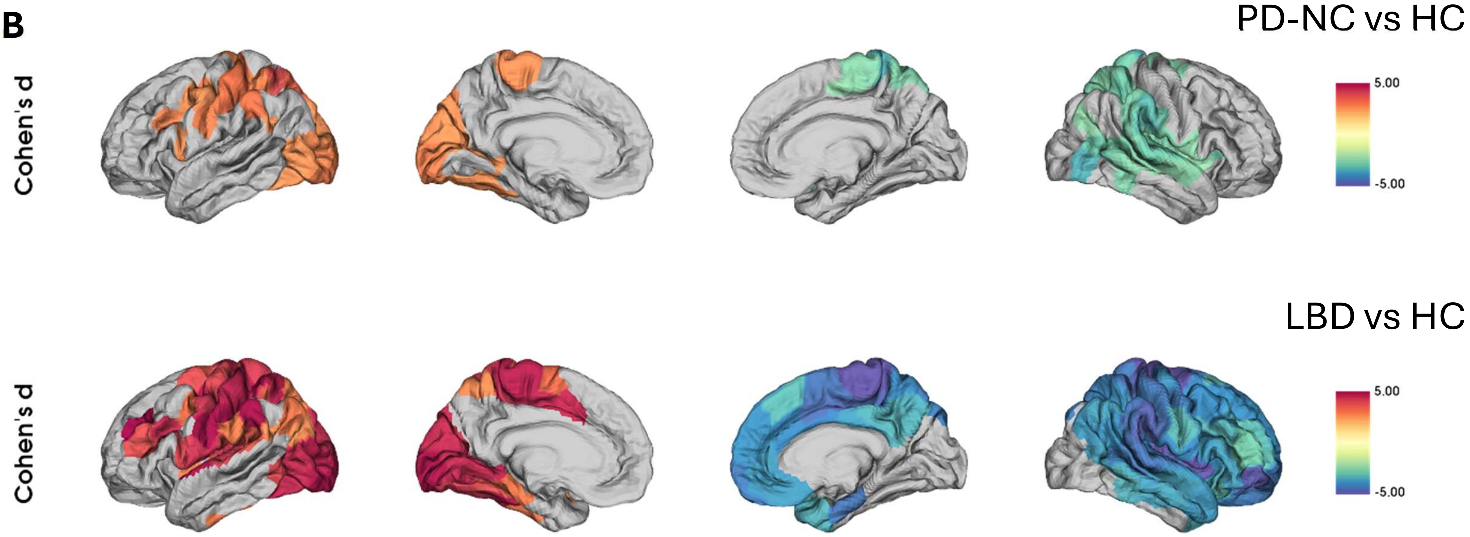
PD-NC vs HC
Cohen's d
5.00
-5.00
LBD vs HC
Cohen's d
5.00
-5.00

C

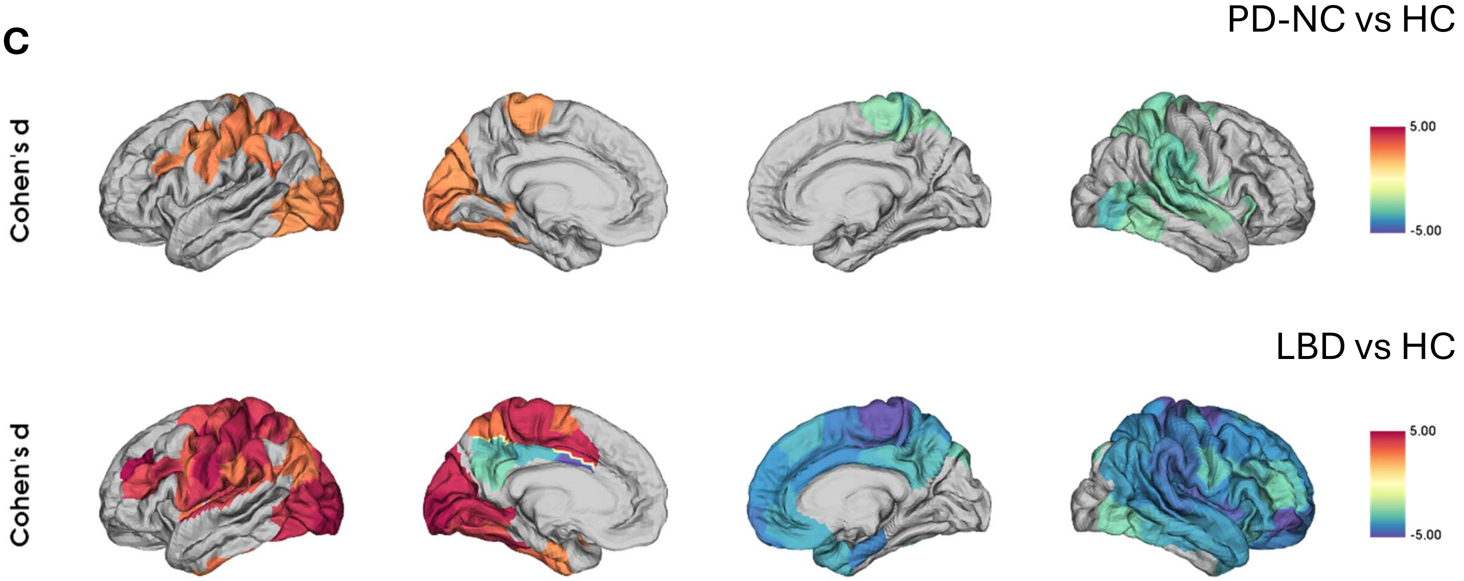
PD-NC vs HC
Cohen's d
5.00
-5.00
LBD vs HC
Cohen's d
5.00
-5.00

Supplementary Figure 7. Differences in inter-regional differentiation between patients with Lewy Body Dementias (LDB), patients with Parkinson's and normal cognition (PD-NC) and controls (HC) – Replication using 7T Quantitative MRI

We calculated mean values for each mutiparameter map (MPM) within 4 regions of interest (ROIs). These were chosen based on their rating on the first primary structural connectivity gradient (SC-G1): two were from the extremes of the gradient distribution ("RH_SalVentAttn_TempOccPar_3", and "RH_SomMot_18") and two from the middle of the gradient distribution ("RH_Default_Temp_1", "LH_Default_Temp_1")).

We compared ROI*Group interaction using mixed linear models with age and sex as covariates to assess whether interregional differences in MPM signal differed significantly between groups.

There was a significant ROI*Group interaction for MTsat (p=0.022) (**A**) and R1 (p=0.045) (**B**) but not proton density (**C**) or R2* (**D**). For MTsat, the group difference in inter-regional MTsat values was driven by LBD in the "RH_SomMot_18" region (β=-0.131, p=0.003). There were no significant pairwise regions for R1.

A

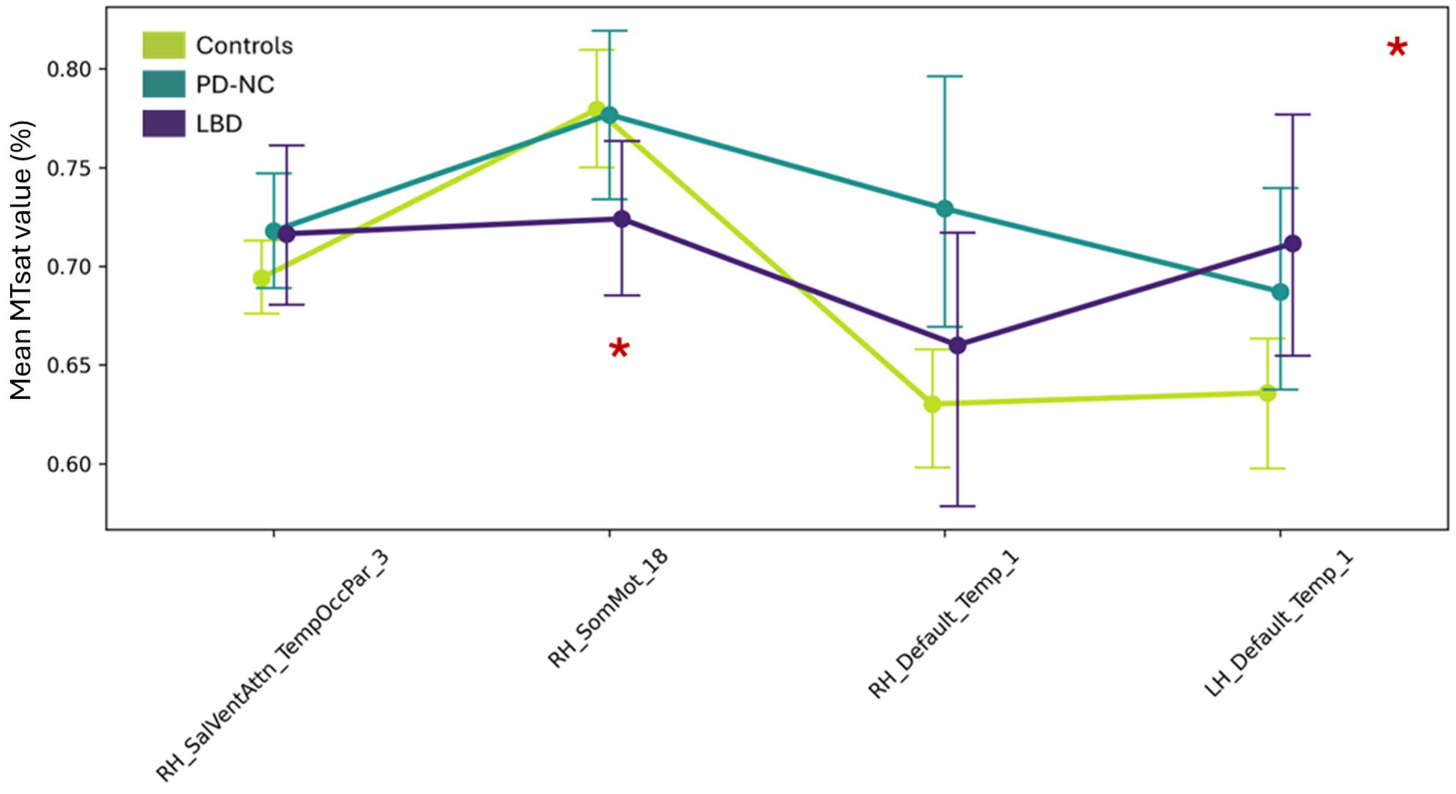

B

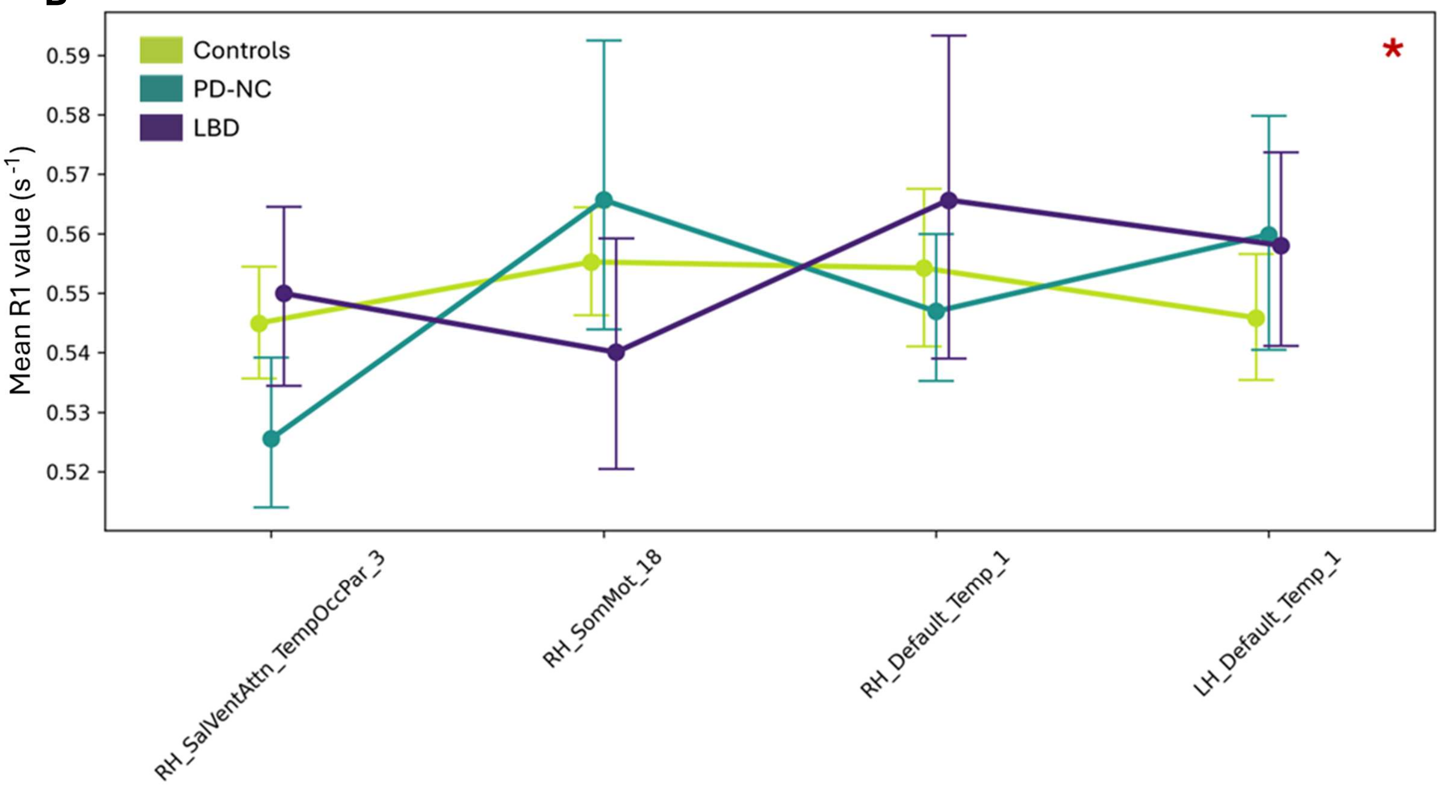
Controls
PD-NC
LBD
*
Mean R1 value (s^-1)
0.59
0.58
0.57
0.56
0.55
0.54
0.53
0.52
RH_SalVentAttn_TempOccPar_3
RH_SomMot_18
RH_Default_Temp_1
LH_Default_Temp_1

C

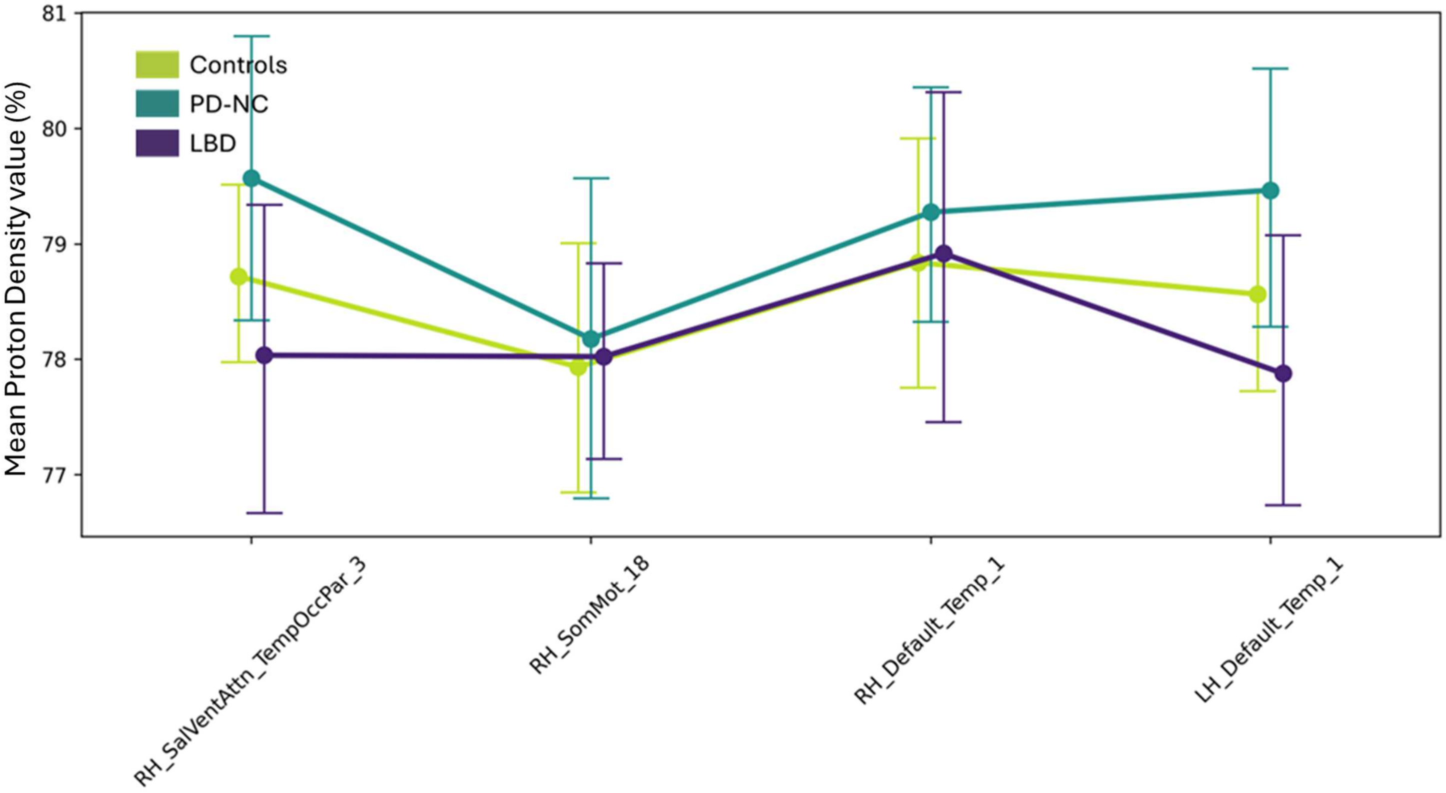
Controls
PD-NC
LBD
Mean Proton Density value (%)
81
80
79
78
77
RH_SalVentAttn_TempOccPar_3
RH_SomMot_18
RH_Default_Temp_1
LH_Default_Temp_1

D

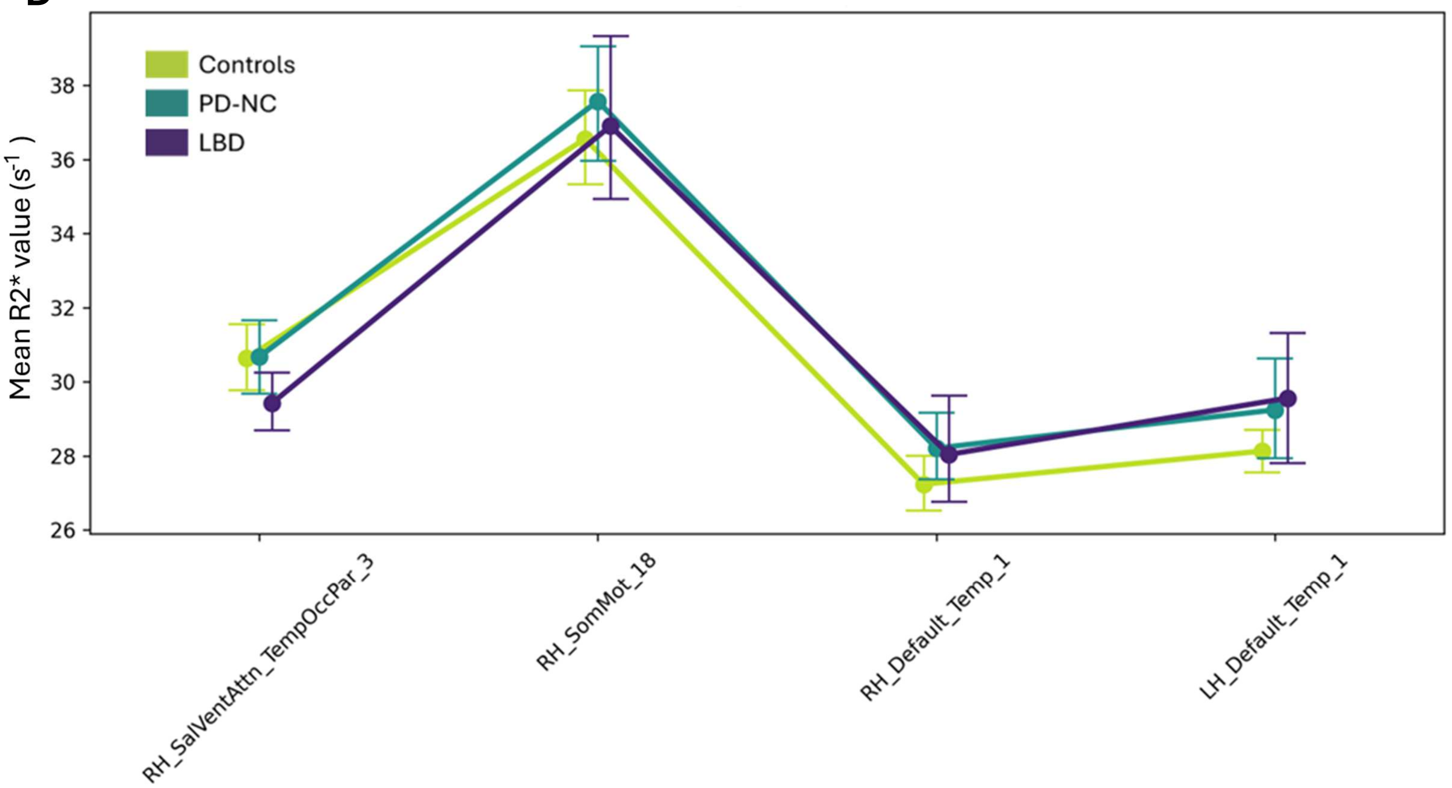
Controls
PD-NC
LBD
Mean R2* value (s⁻¹)
38
36
34
32
30
28
26
RH_SalVentAttn_TempOccPar_3
RH_SomMot_18
RH_Default_Temp_1
LH_Default_Temp_1

Supplementary Figure 8. Differences in composite gradient difference score between LBD subgroups.

To ensure that no particular LBD subgroup was driving our results regarding the correlation of overall gradient change with measures of clinical severity, we assessed whether composite gradient difference scores significantly differed between the different LBD subgroups: Parkinson's with mild cognitive impairment (PD-MCI, n=4), Parkinson's dementia (PDD, n=22), Dementia with Lewy Bodies (DLB, n=36).

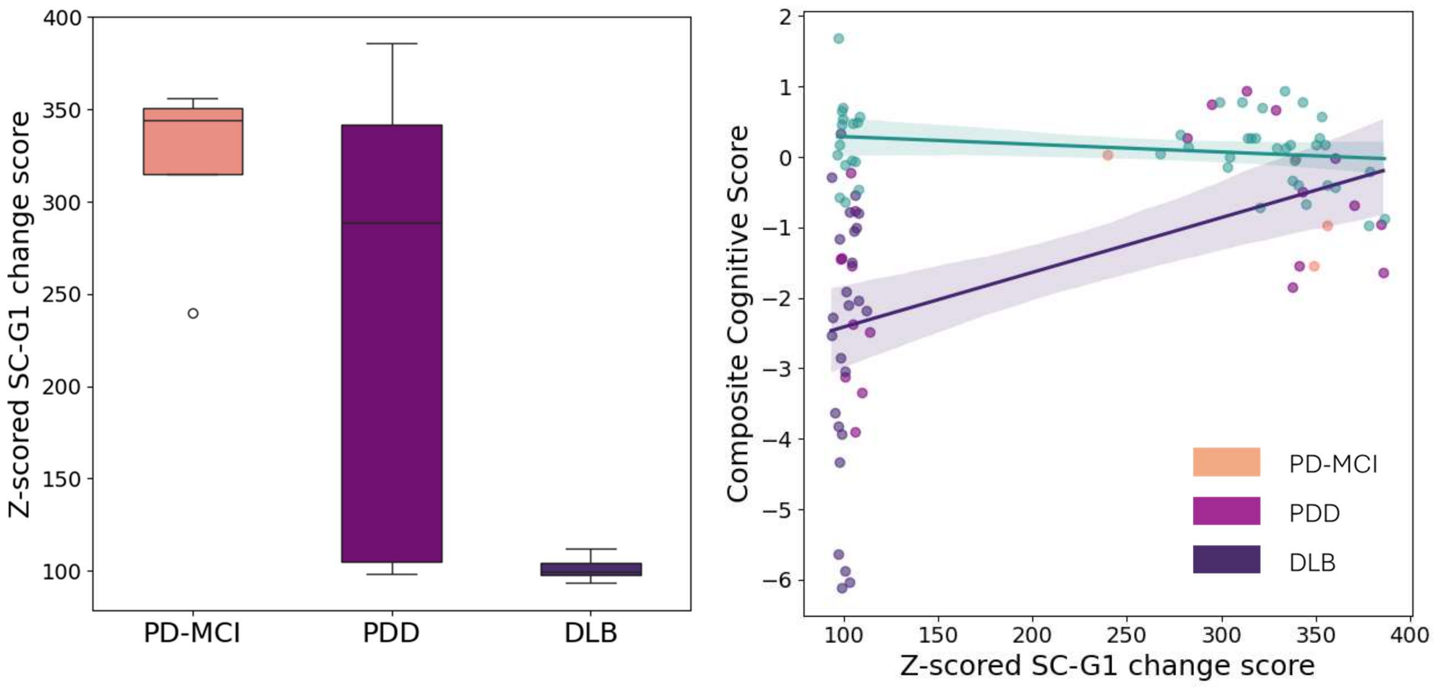

We found that although overall the groups differed (Kruskal Wallis W=27.1, p<0.001) there were no significant differences between any of the two groups in post-hoc testing (all individual comparisons showed p>0.39). The relationship between composite cognitive score and Z-scored SC-G1 change score was driven by the less affected PD-MCI and PDD participants who showed more overall gradient changes than controls; in contrast, more affected DLB and PDD participants had lower gradient change scores (more similar overall gradient distribution to that of control participants).

The 15 LBD participants that showed the highest Z-scored SG-G1 change score did not differ form the other LBD participants in demographics or motor characteristics:

**Characteristic**	**Statistic**	**p-value**
Age	H=1.90	0.168
Sex	x^2=0	1.0
Years education	H= 0.80	0.371
Years diagnosis	H =1.98	0.159
UPDRS total	H=1.86	0.173
UPDRS motor	H=0.10	0.752
LEED	H=0.43	0.510

Supplementary Figure 9. Structural gradient alterations in Lewy body disease are associated with regional gene expression patterns

We assessed whether principal structural gradient alterations in patients with Lewy body dementia (LBD) and Parkinson's with intact cognition (PD-NC) are underpinned by normative differences in genes related to specific biological processes and pathways. We performed partial least squares regression (PLS) with dependent variable Y the t-map of primary structural gradient (SC-G1) alterations (1*200 regions, LBD vs HC and PD vs HC assessed separately) and predictor matrix X of regional gene expression (17545*200 regions derived from the Allen Human brain atlas[18]).

The first principal component (PLS1), explaining most variance in both gene expression and SC-G1 variability was used in further analyses. The regional profile of PLS1 weighting for LBD vs GC **(A)** reveals that left parietal and frontal regions showed most downweighting (reduced expression) of genes. Genes that were significantly downweighted (against 1000 spatially correlated spin-permutations, $p_{spin}<0.05$) were then included in gene ontology (GO) and enrichment analyses. Down-weighted genes, that were less expressed in regions showing SC-G1 differences in LBD vs HC, were enriched for GO terms relating to cellular response to stimulus, positive regulation of biological and cellular processes, and developmental processes **(B)** with similar results for down-weighted genes in PD-NC vs HC **(C)**. Although GO terms for LBD and PD-NC were highly inter-correlated, there were several terms that were solely enriched in LBD and not in PD-NC **(D)** or only enriched in PD-NC and not in LBD patients **(E)**.

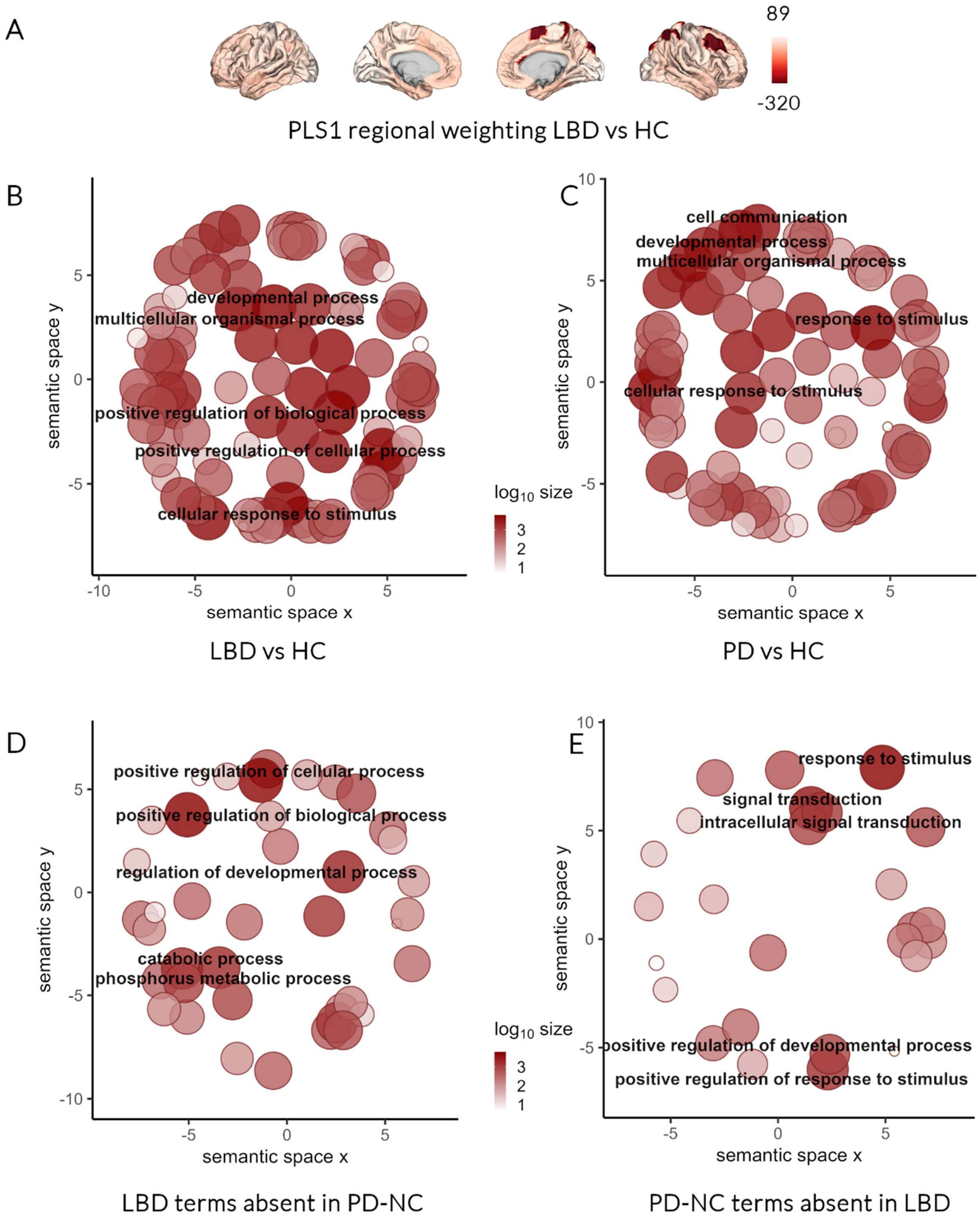
A
89
-320
PLS1 regional weighting LBD vs HC
B
developmental process
multicellular organismal process
positive regulation of biological process
positive regulation of cellular process
cellular response to stimulus
semantic space x
semantic space y
log10 size
LBD vs HC
C
cell communication
developmental process
multicellular organismal process
response to stimulus
cellular response to stimulus
semantic space x
semantic space y
PD vs HC
D
positive regulation of cellular process
positive regulation of biological process
regulation of developmental process
catabolic process
phosphorus metabolic process
semantic space x
semantic space y
log10 size
LBD terms absent in PD-NC
E
response to stimulus
signal transduction
intracellular signal transduction
positive regulation of developmental process
positive regulation of response to stimulus
semantic space x
semantic space y
PD-NC terms absent in LBD

References

1. Parkes, L., Fulcher, B., Yücel, M. & Fornito, A. An evaluation of the efficacy, reliability, and sensitivity of motion correction strategies for resting-state functional MRI. *NeuroImage* **171**, 415–436 (2018).

2. Esteban, O. *et al.* MRIQC: Advancing the automatic prediction of image quality in MRI from unseen sites. *PloS One* **12**, e0184661 (2017).

3. Schaefer, A. *et al.* Local-Global Parcellation of the Human Cerebral Cortex from Intrinsic Functional Connectivity MRI. *Cereb. Cortex N. Y. N 1991* **28**, 3095–3114 (2018).

4. Tournier, J.-D. *et al.* MRtrix3: A fast, flexible and open software framework for medical image processing and visualisation. *NeuroImage* **202**, 116137 (2019).

5. Veraart, J., Fieremans, E. & Novikov, D. S. Diffusion MRI noise mapping using random matrix theory. *Magn. Reson. Med.* **76**, 1582–1593 (2016).

6. Kellner, E., Dhital, B., Kiselev, V. G. & Reisert, M. Gibbs-ringing artifact removal based on local subvoxel-shifts. *Magn. Reson. Med.* **76**, 1574–1581 (2016).

7. Anderson, G. Assuring Quality/Resisting Quality Assurance: Academics' responses to 'quality' in some Australian universities. *Qual. High. Educ.* **12**, 161–173 (2006).

8. Tustison, N. J. *et al.* N4ITK: improved N3 bias correction. *IEEE Trans. Med. Imaging* **29**, 1310–1320 (2010).

9. Modat, M. *et al.* Fast free-form deformation using graphics processing units. *Comput. Methods Programs Biomed.* **98**, 278–284 (2010).

10. Smith, R. E., Tournier, J.-D., Calamante, F. & Connelly, A. Anatomically-constrained tractography: improved diffusion MRI streamlines tractography through effective use of anatomical information. *NeuroImage* **62**, 1924–1938 (2012).

11. Tournier, J.-D., Calamante, F. & Connelly, A. Improved probabilistic streamlines tractography by 2nd order integration over fibre orientation distributions | Request PDF. *Proc. Int. Soc. Magn. Reson. Med.* 1670 (2010).

12. Smith, R. E., Tournier, J.-D., Calamante, F. & Connelly, A. SIFT2: Enabling dense quantitative assessment of brain white matter connectivity using streamlines tractography. *NeuroImage* **119**, 338–351 (2015).

13. Esteban, O. *et al.* fMRIPrep: a robust preprocessing pipeline for functional MRI. *Nat. Methods* **16**, 111–116 (2019).

14. Jenkinson, M., Bannister, P., Brady, M. & Smith, S. Improved Optimization for the Robust and Accurate Linear Registration and Motion Correction of Brain Images. *NeuroImage* **17**, 825–841 (2002).

15. Andersson, J. L. R., Skare, S. & Ashburner, J. How to correct susceptibility distortions in spin-echo echo-planar images: application to diffusion tensor imaging. *NeuroImage* **20**, 870–888 (2003).

16. Greve, D. N. & Fischl, B. Accurate and robust brain image alignment using boundary-based registration. *NeuroImage* **48**, 63–72 (2009).

17. Blauwendraat, C., Nalls, M. A. & Singleton, A. B. The genetic architecture of Parkinson's disease. *Lancet Neurol*. **19**, 170–178 (2020).

18. Hawrylycz, M. *et al*. Canonical genetic signatures of the adult human brain. *Nat. Neurosci*. **18**, 1832–1844 (2015).
