# Supplementary material for: Evidence for divergent cortical organisation in Parkinson’s disease and Lewy Body Dementia
Source: Nat Commun. 2025 Nov 25;16:11623. doi: 10.1038/s41467-025-66783-9 (PMC12749319; doi:10.1038/s41467-025-66783-9)
Supplement: Supplementary file 2 — Reporting Summary [file 41467_2025_66783_MOESM2_ESM.pdf]

nature portfolio

Corresponding author(s): Dr Angeliki Zarkali

Last updated by author(s): Jul 15, 2025

Reporting Summary

Nature Portfolio wishes to improve the reproducibility of the work that we publish. This form provides structure for consistency and transparency in reporting. For further information on Nature Portfolio policies, see our Editorial Policies and the Editorial Policy Checklist.

Statistics

For all statistical analyses, confirm that the following items are present in the figure legend, table legend, main text, or Methods section.

n/a	Confirmed	
☐	☒	The exact sample size (*n*) for each experimental group/condition, given as a discrete number and unit of measurement
☐	☒	A statement on whether measurements were taken from distinct samples or whether the same sample was measured repeatedly
☐	☒	The statistical test(s) used AND whether they are one- or two-sided<br>*Only common tests should be described solely by name; describe more complex techniques in the Methods section.*
☐	☒	A description of all covariates tested
☐	☒	A description of any assumptions or corrections, such as tests of normality and adjustment for multiple comparisons
☐	☒	A full description of the statistical parameters including central tendency (e.g. means) or other basic estimates (e.g. regression coefficient) AND variation (e.g. standard deviation) or associated estimates of uncertainty (e.g. confidence intervals)
☐	☒	For null hypothesis testing, the test statistic (e.g. *F*, *t*, *r*) with confidence intervals, effect sizes, degrees of freedom and *P* value noted<br>*Give P values as exact values whenever suitable.*
☒	☐	For Bayesian analysis, information on the choice of priors and Markov chain Monte Carlo settings
☒	☐	For hierarchical and complex designs, identification of the appropriate level for tests and full reporting of outcomes
☐	☒	Estimates of effect sizes (e.g. Cohen's *d*, Pearson's *r*), indicating how they were calculated

Our web collection on statistics for biologists contains articles on many of the points above.

Software and code

Policy information about availability of computer code

Data collection	Demographic, clinical and psychometric data was collected and stored using Redcap or Excel. MRI data was collected using a 3T and 7T Siemens scanner and preprocessed using Mrtrix 3.0 for diffusion data, Nipype for resting state fmri data and hMRI toolbox for multiparametric map generation. All steps of data processing are described in the manuscript.
Data analysis	Gradients were derived using BrainSpace v1.0. Statistical analyses were performed in Python 3. All analyses are described in full in the manuscript. All analysis code will be made available upon publication: https://github.com/AngelikaZa/RegionalDifferentiationLBD.

For manuscripts utilizing custom algorithms or software that are central to the research but not yet described in published literature, software must be made available to editors and reviewers. We strongly encourage code deposition in a community repository (e.g. GitHub). See the Nature Portfolio guidelines for submitting code & software for further information.

Data

Policy information about availability of data

All manuscripts must include a data availability statement. This statement should provide the following information, where applicable:

- Accession codes, unique identifiers, or web links for publicly available datasets
- A description of any restrictions on data availability
- For clinical datasets or third party data, please ensure that the statement adheres to our policy

All data and results of statistical analyses are presented in the manuscript. All analysis code will be made available upon publication: https://github.com/AngelikaZa/RegionalDifferentiationLBD. Patient level data may be shared upon reasonable request to the corresponding author.

Research involving human participants, their data, or biological material

Policy information about studies with human participants or human data. See also policy information about sex, gender (identity/presentation), and sexual orientation and race, ethnicity and racism.

Reporting on sex and gender	Information on sex is reported in Table 1. All analyses were corrected for age and sex.
Reporting on race, ethnicity, or other socially relevant groupings	Ethnicity data was not collected during this study therefore we are not able to report this. We report data on age, sex, handedness, and years of education in Table 1. All analyses were corrected for age and sex.
Population characteristics	Two cohorts are included. For our main cohort (3T) a total of 131 participants were included: 62 patients with Lewy Body Dementia (including Dementia with Lewy Bodies, Parkinson's disease dementia and Parkinson's with mild cognitive impairment), 46 Parkinson's patients with intact cognition (PD-NC) and 23 healthy age-matched controls. For our replication 7T cohort, 58 participants were recruited: 13 with LBD, 19 PD-NC and 23 age-matched controls.
Recruitment	For both 3T and 7T cohorts, participants were recruited to University College London (UCL). Participants were recruited for cognitive and movement disorders clinic in affiliated hospitals or from patient support groups.
Ethics oversight	All participants provided written informed consent, and the study was approved by the Queen Square Research Ethics Committee (15.LO.0476).

Note that full information on the approval of the study protocol must also be provided in the manuscript.

Field-specific reporting

Please select the one below that is the best fit for your research. If you are not sure, read the appropriate sections before making your selection.

[x] Life sciences [ ] Behavioural & social sciences [ ] Ecological, evolutionary & environmental sciences

For a reference copy of the document with all sections, see nature.com/documents/nr-reporting-summary-flat.pdf

Life sciences study design

All studies must disclose on these points even when the disclosure is negative.

Sample size	Participants were recruited to the Vision in Parkinson's disease study, a longitudinal observation study of Parkinson's disease. The study was powered to be able to detect differences in high level visual performance at baseline for participants with Parkinson's disease. Additionally, participants were recruited from a cohort study of Dementia with Lewy bodies and Parkinson's dementia; this was powered to detect group differences in imaging and plasma biomarkers between patients with Dementia with Lewy bodies and those with Parkinson's dementia.
Data exclusions	Only participants that passed visual quality control of imaging data for each modality were included. In addition to visual inspection, we adopted strict motion-control criteria for 3T rsfMRI, given susceptibility to motion artefact1. Specifically, participants were excluded if any of the following was met: 1) mean frame-wise displacement (FD) >0.3mm, 2) any FD >5mm, or 3) outliers >30% of the whole sample. FD was calculated using the MRI Quality Control tool (MRIQC)2. This led to 23 participants being excluded from functional connectivity analyses (11 PD-NC and 12 LBD), resulting to a total of 111 participants included in functional connectivity analyses (n=23 controls, n=35 PD-NC, and n=50 LBD).<br>For the replication cohort, of 65 participants (n=24 controls, n=23 PD and n=20 LBD) with acquired MPM and MP2RAGE data, 8 participants (n=4 PD and n=4 LBD) were excluded due to significant artefact on the acquired multiparametric maps, and 1 LBD participant was excluded due to artefact on MP2RAGE. This resulted in a total of 58 participants (n=24 controls, n=19 PD-NC and 15 LBD) included. Additionally, of the included participants at 7T, 3 (n=1 control, n=2 LBD) did not have MTsat maps acquired, leaving n=23 controls, n=19 PD-NC and 13 LBD participants in MTsat analyses.
Replication	To ensure the robustness of our results, we replicated our findings in a separate cohort of LBD, PD-NC and controls using different MR acquisitions and analyses. We used 7 Tesla quantitative MRI (7T qMRI) to test qMRI values from regions at the extremes of SG-G1 distribution (that differed between groups in our main analysis) against regions from the middle of the distribution (which did not differ between groups). We included 13 LBD, 19 PD-NC and 23 age-matched controls in our replication 7T cohort. Demographics and results of clinical assessments in the replication cohort are presented in Supplementary Table 3. We computed quantitative multiparametric maps (MPM) including proton density, longitudinal relaxation rate (R1), effective transverse relaxation rate (R2*), and magnetisation transfer saturation (MTsat)). We extracted mean MPM values for four ROIs of the Schaeffer parcellation based on their SC-G1 ratings from the main 3T cohort and whether they differed in LBD compared to controls. Thus, two regions were selected from the extremes of the gradient distribution which differed between LBD and controls ("RH_SalVentAttn_TempOccPar_3", and "RH_SomMot_18") and two were selected from the middle of the gradient distribution and which did not show differences between LBD vs controls ("RH_Default_Temp_1", "LH_Default_Temp_1"). We then used mixed linear models accounting for age and sex to study the ROI*Group (LBD, PD-NC, HC) interaction and assess whether interregional differences in MPM values differed significantly between groups.
Randomization	This was an observational study therefore no randomisation took place.
Blinding	The researchers collecting clinical and imaging data were blinded during data collection and MRI quality control. Groups were derived later from participant performance on cognitive tasks and not allocated by the researchers.

Reporting for specific materials, systems and methods

We require information from authors about some types of materials, experimental systems and methods used in many studies. Here, indicate whether each material, system or method listed is relevant to your study. If you are not sure if a list item applies to your research, read the appropriate section before selecting a response.

Materials & experimental systems

n/a	Involved in the study
☒	☐ Antibodies
☒	☐ Eukaryotic cell lines
☒	☐ Palaeontology and archaeology
☒	☐ Animals and other organisms
☐	☒ Clinical data
☒	☐ Dual use research of concern
☒	☐ Plants

Methods

n/a	Involved in the study
☒	☐ ChIP-seq
☒	☐ Flow cytometry
☐	☒ MRI-based neuroimaging

Clinical data

Policy information about clinical studies
All manuscripts should comply with the ICMJE guidelines for publication of clinical research and a completed CONSORT checklist must be included with all submissions.

Clinical trial registration: REC reference: 15/LO/0476

Study protocol: The study protocol and other information about the study can be found here: https://vision-in-parkinsons.co.uk/

Data collection: All clinical and imaging data was collected over a single study visit. Clinical and psychological assessments were performed at the Institute of Neurology, Queen Square, University College London. All imaging data was collected at the same scanner (3T Siemens) at the Department for Advanced Neuroimaging, 12 Queen Square, University College London.

Outcomes: This was an observational study. Participants with cognitive impairment were classified as LBD: including patients diagnosed with Dementia with Lewy bodies (according to the McKeith diagnostic criteria), or Parkinson's dementia (defined as an established clinical diagnosis of Parkinson's disease and dementia or impaired function in activities of daily leaving (impaired functional assessments questionnaire) and a Montreal Cognitive Assessment (MoCA) score below 26) or Parkinson's disease with Mild cognitive impairment defined as a clinical diagnosis of PD and persistent performance below 1.5 standard deviations (SD) in at least two different tests in one cognitive domain or one cognitive test in at least two cognitive domains, according to respective MDS clinical criteria. All remaining participants with Parkinson's disease were classified as PD-NC.

Plants

Seed stocks: N/A

Novel plant genotypes: *Describe the methods by which all novel plant genotypes were produced. This includes those generated by transgenic approaches, gene editing, chemical/radiation-based mutagenesis and hybridization. For transgenic lines, describe the transformation method, the number of independent lines analyzed and the generation upon which experiments were performed. For gene-edited lines, describe the editor used, the endogenous sequence targeted for editing, the targeting guide RNA sequence (if applicable) and how the editor was applied.*

Authentication: *Describe any authentication procedures for each seed stock used or novel genotype generated. Describe any experiments used to assess the effect of a mutation and, where applicable, how potential secondary effects (e.g. second site T-DNA insertions, mosiacism, off-target gene editing) were examined.*

Magnetic resonance imaging

Experimental design

Design type: Resting state

Design specifications: N/A

Behavioral performance measures: N/A

Acquisition

Imaging type(s)	Resting state FMRI
Field strength	3T
Sequence & imaging parameters	All MRI data were acquired on a 3T Siemens Magnetom Prisma scanner (Siemens) with a 64-channel head coil. Resting state functional MRI (rsfMRI) was acquired with the following parameters: gradient-echo EPI, TR=70ms, TE=30ms, flip angle=90°, FOV=192×192, voxel size=3x3x2.5 mm, 105 volumes, 7-minute session. During rsfMRI, participants were instructed to lie quietly with their eyes closed and avoid falling asleep; this was confirmed by monitoring and post-scan debriefing. A 3D MPRAGE (magnetization prepared rapid acquisition gradient echo) image (voxel size=1×1×1 mm, TE=3.34ms, TR= 2530 ms, flip angle=7°) was also obtained. Imaging for all participants was performed at the same time of day, with PD participants receiving their normal medications.
Area of acquisition	Whole brain scans were acquired

Diffusion MRI ☒ Used ☐ Not used

Parameters: Diffusion weighted imaging (DWI) was acquired with the following parameters: b0 in both AP and PA directions, b=50 s/mm2 /17 directions, b=300 s/mm2 /8 directions, b=1000 s/mm2 /64 directions, b=2000 s/mm2 /64 directions, 2x2x2 mm isotropic voxels, TE=3260ms, TR=58ms, 72 slices, 2mm thickness, acceleration factor = 2. DWI acquisition time was approximately 10 min.

Preprocessing

Preprocessing software	Pre-processing of DWI images was performed in MRtrix3.0.<br>rsfMRI data underwent standard pre-processing using fMRIPrep 23.2.0.
Normalization	The raw T1-weighted images were then registered to the diffusion-weighted image (rigid, affine transformation) using Niftyreg.
Normalization template	The Schaefer parcellation was used to generate 200 cortical regions of interest (ROIs) by segmenting each participant's T1-weighted image. The same parcellation was used both for structural and functional connectome construction.
Noise and artifact removal	DWI weighted images were preprocessed using the standard pipeline as implemented in MRtrix3.0. This involves denoising, removal of Gibbs artefacts, eddy-current and motion artefact correction and bias field correction. Five tissue anatomical segmentation was performed in native DWI-image space using the 5ttgen script in MRtrix. Anatomically-constrained tractography was then performed with 10 million streamlines, using the iFOD2 algorithm and dynamic seeding with streamlines truncated at the interface of grey-white matter. Spherical deconvolution informed filtering of tractograms (SIFT2) algorithm was then applied to reduce biases. Resulting streamlines were used to construct the structural connectome, weighted by streamline count and a cross-sectional area multiplier.<br>Functional connectivity between ROIs was quantified as the Pearson correlation coefficient between mean regional BOLD time series resulting to a 200x200 undirected weighted connectivity matrix.
Volume censoring	N/A

Statistical modeling & inference

Model type and settings	Gradient scores were compared between groups (PD-NC vs HC, LBC vs HC and LBD vs PD-NC) using surface-based linear models implements in BrainStat, with age and sex as covariates and family-wise error (FWE) correction using random field theory and the default cluster-defining threshold 0.01.
Effect(s) tested	We correlated regional differences between PD-NC vs HC, LBD vs HC and LBD vs PD-NC. We contextualised the differences in structural gradients found in LBD and PD-NC compared to HC with respect to normative variations in 1) excitatory and inhibitory neuronal gene expression markers, 2) cortical cytoarchitecture, 3) disease specific genetic and plasma markers, and 4) global gene expression. This was done using the unthresholded t-map of differences in structural gradients LBD vs HC and PD vs HC), corrected for age and sex, was parcellated (Shaefer 200 cortical regions) using the neuromaps Parcellater tool. We compared the parcellated t-map of gradient differences to different contextual measures using spearman correlation and spin permutation tests (1000 permuations, pspin <0.05).

Specify type of analysis: ☐ Whole brain ☐ ROI-based ☒ Both

Anatomical location(s): 200 cortical regions of interest (ROIs) were generated by segmenting each participant's T1-weighted image using the Schaefer parcellation.

Statistic type for inference<br>(See Eklund et al. 2016)	Surface-based general linear models
Correction	Family-wise error (FWE) correction using random field theory and the default cluster-defining threshold 0.01.

Models & analysis

n/a	Involved in the study
☐	☒ Functional and/or effective connectivity
☒	☐ Graph analysis
☒	☐ Multivariate modeling or predictive analysis

Functional and/or effective connectivity	Functional connectivity between ROIs was quantified as the Pearson correlation coefficient between mean regional BOLD time series.
